# What factors influence midwives to provide obstetric high dependency care on the delivery suite or request care be escalated away from the obstetric unit? Findings of a focus group study

**DOI:** 10.1186/s12884-019-2487-0

**Published:** 2019-09-09

**Authors:** Alison James, Simon Cooper, Elizabeth Stenhouse, Ruth Endacott

**Affiliations:** 1 Faculty of Health and Human Sciences, School of Nursing and Midwifery, University of Plymouth, Drake Circus, Plymouth, Devon PL4 8AA UK; 2 School of Nursing and Healthcare Professions, Federation University, Ballarat, Australia

**Keywords:** Obstetric high dependency care, Escalation of care, Maternal critical care

## Abstract

**Background:**

In the United Kingdom, midwives will engage in discussions with the multidisciplinary team as to whether they can provide Obstetric High Dependency Care (OHDC) on the Delivery Suite or whether a woman’s care should be escalated to the critical care team. This study aimed to explore the question: What factors influence midwives to provide OHDC or request care be escalated away from the obstetric unit in hospitals remote from tertiary referral centres?

**Methods:**

Focus groups were undertaken with midwives (*n* = 34) across three obstetric units in England, with annual birth rates ranging from 1500 to 5000 per annum, in District General Hospitals. Three scenarios in the form of video vignettes of handover were used as triggers for the focus groups. Scenario 1; severe pre-eclampsia, physiologically unstable 2; major postpartum haemorrhage requiring invasive monitoring 3; recent admission of woman with chest pain receiving facial oxygen and requiring continuous electrocardiogram (ECG) monitoring. Two focus groups were conducted in each of the obstetric units with experienced midwives. Data were analysed using a qualitative framework approach.

**Results:**

Factors influencing midwives’ care escalation decisions included the care environment, a woman’s diagnosis and fetal or neonatal factors. The overall plan of care including the need for ECG and invasive monitoring were also influential factors. Midwives in the smallest obstetric unit did not have access to the facilities for OHDC provision. Midwives in the larger obstetric units provided OHDC but identified varying degrees of skill and sometimes used ‘workarounds’ to facilitate care provision. Midwifery staffing levels, skill mix and workload were also influential. Some differences of opinion were evident between midwives working in the same obstetric units as to whether OHDC could be provided and the support they would enlist to help them provide it. Reliance on clinical guidelines appeared variable.

**Conclusions:**

Findings indicate that there may be inequitable OHDC provision at a local level. Organisationally robust systems are required to promote safe, equitable OHDC care including skills development for midwives and precise escalation guidelines to minimise workarounds. Training for midwives must include strategies that prevent skills fade.

## Background

### Introduction

Women who become acutely ill during pregnancy or the intrapartum / postnatal periods may be transferred to a critical care unit for complex treatments including organ system monitoring and support [1, 2] and some will remain in the Obstetric Unit (OU) and receive Obstetric High Dependency Care (OHDC) [3, 4] on the Delivery Suite. Obstetric High Dependency Care has been identified as a vital aspect of maternity care in the United Kingdom (UK) given the increasing numbers of women who present with complex pregnancies because of comorbidities or obstetric complications [3, 5]. Research findings and expert opinion suggest that local variations exist in the definition of OHDC and terminology changes with time [6–11]. OHDC has been defined as;

*“an interim level of care for women requiring interventions over and above the [specialised] ‘high risk’ obstetric care that will be carried out routinely on a consultant led labour ward … It will be implemented where a woman has deteriorated clinically but her care can be managed appropriately on the labour ward”* [10].

In the UK, midwives will engage in discussions with the multidisciplinary team as to whether they can provide OHDC on the Delivery Suite or a woman requires her care to be escalated [12]. Escalation of care is defined by Posner and Freund (2004: p 438) as *“Any significant unplanned increase in the level of care provided to the patient and includes such outcomes as unplanned intensive care unit admission”* [13]. This focus group study examined the factors that influence midwives working in OUs remote from tertiary referral centres to provide OHDC or request a woman’s care be escalated away from the OU. Tertiary referral centres are those OUs classed as regional or national centres of excellence, providing specialist care for women with for example, complex comorbidities, and this sets them aside from District General hospitals in the UK [14].

### Indications for OHDC

A UK retrospective survey of OHDC provision in a tertiary referral OU found that 50% of women were admitted for obstetric haemorrhage, 16% for hypertensive disorders and 10% for cardiac disorders [15]. The researchers conducting this survey acknowledge the findings may not be generalisable to all OUs because there is likely to be a higher prevalence of OHDC in tertiary referral centres due to the specialist nature of the care provided [15]. Overall, there are indications that more women receive OHDC for obstetric reasons than comorbidities alone, [15, 16] although the retrospective nature of some of the studies may have an impact on the quality of the data [17].

### Identifying women who require OHDC

Healthcare professionals must demonstrate a wide range of skills including sound clinical decision-making, communication and team working in order to recognise and manage the deteriorating woman [18, 19]. However, additional measures are in place to identify those women who require additional monitoring and clinical interventions. Rapid Response Systems (RSS) include strategies to detect clinical deterioration and trigger a response (Track and Trigger Systems (TTS)) and strategies to respond to the clinical deterioration (Escalation of Care (EoC) guidelines) [20–22]. RRS have been widely evaluated in terms of their impact on hospital mortality and cardiac arrest rates and have been shown to reduce hospital mortality rates (RR 0.87, 95% CI 0.81–0.95, *P* < 0.001) [23].

A number of different TTS are available, including those specifically designed for obstetric patients termed Modified Early Obstetric Warning Systems (MEOWS) [24, 25]; these work by assigning scores to clinical observations that can be routinely recorded in the ward environment. Scores rise as deviations away from normal physiological parameters occur and predetermined scores act as the trigger for professionals to initiate the appropriate referral and treatment in line with EoC guidelines [22, 24, 25]. Escalation of care guidelines also provide local direction as to when specialists such as the Critical Care Outreach Team or Medical Emergency Team should be involved in the care of an acutely ill woman, or her care be transferred to a specialist clinical area such as the ICU or OHDC be provided [2].

There is robust evidence to suggest that when EoC guidelines are not followed, failure to rescue situations occur [26]. The term ‘*failure to rescue’* is used to identify when patient mortality or morbidity has occurred because one or more aspects of the escalation process has failed [27–29]. Reasons for failure to rescue may include inaccurate completion of TTS, poor reporting in accordance with the EoC guideline [26, 28, 30] and during periods of high clinical activity, patient monitoring and completion of TTS systems may be interrupted or prioritised as being of low importance when compared with other clinical responsibilities [26, 31].

### Obstetric high dependency care provision

Smaller OUs may not have the necessary resources or pool of clinical expertise to form a “specialized team” to care for women needing higher care levels [7]. Moreover, Scrutton and Gardner (2012) theorise that women requiring high dependency care in ‘small’ OUs are more likely to be transferred to ICU, but do not clarify what constitutes small in terms of the annual birth rate and do not justify this proposition [6]. Correspondingly, low level evidence suggests that tertiary referral centres are more likely to provide OHDC than District General Hospitals, especially those that have lower birth rates [15, 16, 32]. These variations are likely to be influenced not only by an OU’s annual birth rate, but characteristics of the local case mix, specialist services offered (such as fetal and maternal medicine), and criteria for transfer of women to critical care units [3, 9, 10, 33].

### Midwives and OHDC

Midwives’ education and training equips them with specialist knowledge and skills including; an in depth understanding of the physiological / psychological changes occurring during pregnancy and the puerperium, the ability to monitor fetal wellbeing, detect abnormalities such as postnatal uterine atony and support breastfeeding [34]. The aforementioned skills may be less familiar to critical care nurses [35, 36] and consequently, OHDC involving midwives may assist in promoting ‘holistic care’ for the mother, neonate / family and enhance continuity of care by reducing transfers away from the OU [16, 37, 38]. Nevertheless, it is also acknowledged that qualified midwives may require post-registration education to afford them the necessary skills (e.g. management of invasive monitoring) to provide OHDC [33, 39–41].

The increase in numbers of midwives who undertake direct entry midwifery programmes (which require no previous professional nursing qualification), is raised as a concern when OHDC provision is discussed [8, 42, 43]. The Midwives in Teaching (MINT) study commissioned by the UK Nursing and Midwifery Council (NMC) identified that newly qualified midwives would have preferred more educational input regarding the care of women with high risk pregnancies and those requiring OHDC during their training [44].

A qualitative study conducted in 2007 identified that midwives felt anxious when caring for critically ill women, did not consistently understand the instructions they received from doctors, and some stated they ‘felt out of their depth’ [1]. More recently, a survey involving midwives working on the Delivery Suite of a tertiary referral centre (*n* = 60) identified that 64% felt they did not have adequate knowledge to care for women receiving high dependency care [42]. Another survey of *n* = 137 OUs highlighted that 71% of all OHDC was undertaken by midwives and 24% of these had not received any formal training [45]. These issues may not be unique to the UK; qualitative research undertaken in a New Zealand tertiary teaching hospital reported that midwives sometimes felt ill prepared to provide OHDC and requested specific education and training [38]. Additionally, once midwives have received OHDC training, they face the challenge of maintaining their competence if they do not encounter this cohort of women on a regular basis [39, 41].

### Summary

Midwives are often the first professionals to detect clinical deterioration in a woman’s condition. They will also be involved in the discussions about whether a woman can receive OHDC or her care be escalated away from the Delivery Suite. There is some evidence to suggest that those midwives working in OUs with lower annual birth rates may be less likely to provide OHDC, and there is suggestion that those midwives working in tertiary referral centres are also more likely to provide OHDC. Therefore, the aim of this study was to answer the research question:

*What factors influence midwives to provide OHDC or request care be escalated away from the obstetric unit in hospitals remote from tertiary referral centres?*

## Methods

### Study design and setting

A qualitative design using focus groups addressed the research question. Short video vignettes were used to trigger discussion in focus groups conducted across three OUs in hospitals in England that were geographically remote from tertiary referral centres. These OUs were purposively chosen for their differing annual birth rates, levels of neonatal care facilities and number of fully equipped, designated OHDC rooms (Table 1).

**Table 1:** An overview of the OUs involved in the Focus Group research

Obstetric Unit	Range of births per annum	Neonatal care facilities^a^	Number of delivery beds	Number of OHDC rooms
H	1500–1900	Special Care Unit (SCU)	5	0
I	4000–4500	Local Neonatal Unit (LNU)	9	0, but OHDC equipment available and taken to the bedside
J	Approximately 5000	Neonatal Intensive Care (NICU)	10	1

^a^UK SCUs provide care for neonates who require additional care and possibly some high dependency care, usually around or after 32 weeks gestation. LNUs provide high dependency / short-term intensive care for neonates born around 28–32 weeks gestation. Neonatal intensive care units admit the sickest and most preterm neonates

### Recruitment and sample selection

Two focus groups were conducted in each OU. One focus group involved Band 6 midwives and the other, Band 7 midwives. Band 6 midwives are Registered Midwives who have been qualified at least 1 year and provide clinical care on a regular basis, whilst Band 7 midwives, often termed coordinators or clinical coordinators, have a more clinical managerial role and oversee all Delivery Suite activities.

### Development of video vignettes

To ‘trigger’ the focus group discussions, three simulated clinical situations in the form of video vignettes were used. The video vignettes were based on a previous study examining OHDC [10] and an audit of clinical notes of women whose conditions had triggered clinical incident reporting. Video vignettes were chosen as opposed to written scenarios, as these encourage participants to “draw their own meaning from observations to a greater extent than written vignettes” [46]. Gould (1996) also identifies that using vignettes to promote discussion about a research topic is appropriate where direct observational methods may be problematic or ethically unsound [47].

The video vignette’s depicted midwife to midwife handover of a woman’s care; written information accompanying each video vignette comprised: a brief generic overview of staffing levels and Delivery Suite workloads; observation and fluid balance charts; excerpts of simulated midwifery documentation and blood results. Incorporation of the objective data made the scenarios as comprehensive and as clinically credible as possible, thereby enhancing the face and content validity [17]. Table 2 outlines the key features of each scenario. Scenarios one and two represented women classed as receiving high dependency care and scenario three, classed as a woman with potential for physiological deterioration [2]. The scenario storyboards were scripted by the researcher and ‘acted’ by student midwives / university employees.

**Table 2:** The key features of the three video vignettes used as triggers for the focus groups

	Scenario 1	Scenario 2	Scenario 3
Clinical Picture	Postnatal mother with severe pre eclampsia at 30/40 gestation. Vaginal birth 90 min previously. Neonate transferred to neonatal unit	Postnatal mother who has recently had a primary PPH. On-going management in progress after the initial emergency treatment. Neonate with mother.	Woman 32/40 pregnant with comorbidities (type 2 diabetes and ventricular septal defect repaired in infancy). Raised BMI. Admitted with mild chest pain and low oxygen saturations (88–90%) in air.
	Intravenous magnesium sulphate / intravenous anti- hypertensives in progress	Blood transfusion in progress.	Continuous ECG in progress
	Uncontrolled hypertension	CVP line in situ due to poor peripheral access	Requiring 4 L/min oxygen, via face mask to maintain oxygen saturations at 97%
	Hyperreflexia, 4 beats of clonus	Hourly CVP readings requested to guide fluid replacement	Stable vital signs whilst patient has oxygen therapy in progress, (but at risk of deterioration)
	Headache	Stable pulse and blood pressure. Lochia within normal limits.	Normal CTG, normal fetal movements.
	Blood picture shows HELLP syndrome	Reduced urine output	Differential diagnosis of cardiac event or pulmonary embolism
	Overall, presents with an unstable clinical picture in view of uncontrolled severe hypertension, blood picture and neurological examination findings.	Overall, relatively stable condition, but requiring CVP monitoring.	Currently stable with oxygen therapy in progress but potential for deterioration
Workload	Moderate. All women on the Delivery Suite are in labour – mainly low risk.	High. All but one of the Delivery Suite rooms are occupied however, anticipated that three women will be transferred home / to the post natal ward in the next hour.	Low to moderate. There are empty rooms, mainly low risk women in labour.
Staffing	Correct number and grades of midwives on duty for the maternity unit in question	All band 6 midwives with one band 7 midwife coordinating. One band 6 midwife off sick.	All band 6 midwives (except one newly qualified midwife) on duty with one band 7 midwife coordinating. No staff off sick.

Key:

*CTG* Cardiotocograph

*ECG* Electrocardiogram

*CVP* Central Venous Pressure

*HELLP* Haemolysis, Elevated Liver enzymes, Low Platelets

*PPH* Post Partum Haemorrhage

A panel of six clinicians assessed the content validity of the key features comprising the three scenarios (Hughes and Huby, 2004). For each scenario, nine statements were developed, describing aspects of the scenario. The raters were asked to provide scores for each of the nine statements using a four point Likert type scale (1 = not accurate, 2 = somewhat accurate, 3 = quite accurate, and 4 = highly accurate) [48]. Ratings of 1 or 2 represented ‘content invalid’ or ‘not accurate’ responses and ratings of 3 or 4 were seen as ‘content valid’ or ‘accurate’ [49]. The Item-Content Validity Index (I-CVI) was calculated by summing the number of content valid responses and dividing this score by the total number of raters [49]. I-CVI should be no lower than 0.78 when there are 6 or more raters according to Lynn (1986) [49]. Three items had I-CVI of 0.83 and these items were spread across the three scenarios. The other items all had I-CVI of 1.0.

### Data collection

The aim was to recruit between 6 and 8 midwives for each focus group, reflecting the maximum number of midwives that might be released from clinical duties at any one time. The focus groups were conducted in seminar / meeting rooms within or in very close vicinity of the maternity units, on hospital property. The researcher moderated the focus groups and an assistant moderator was also present and took detailed notes throughout the focus groups (except on one occasion when a focus group was rescheduled at short notice). The midwives were also asked to complete a brief demographic data sheet and individual questionnaires relating to each of the three scenarios that asked ‘What do you want to do in terms of care escalation and why? The organisation of the focus groups and completion of the individual questionnaires were explained to the participants in order to promote parity of data collection processes across the three different hospital sites [50].

The video vignettes were shown to the participants using a portable projector. After the participants had viewed the first video vignette, they were given the supplementary objective data and were asked to complete the individual questionnaire. The focus group for each of the three video vignettes commenced when all the midwives had completed their individual questionnaires. Each focus group was digitally recorded and transcribed verbatim.

### Data analyses

The data were analysed using the framework analytical approach [51, 52]. Inductive reasoning was employed through a process of open coding and deductive reasoning utilised codes that were developed a priori from the categories and subcategories identified during a preceding study examining high dependency care [53]. The data from the first two focus groups and two sets of ‘individual data sheets’ were open coded [52] to enable the researcher to examine the data in a comprehensive manner and ensure that no significant topics or issues were overlooked [52]. ‘A priori’ codes were applied to the data, once the initial inductive open coding process on the subset of data was complete.

Once the framework matrix was complete, the remaining focus group data and midwives’ individual data were coded to the subcategories and categories developed. This process may be described as ‘indexing’ [51] and the use of NVivo, enabled the researcher to organise the data in a systematic manner that enabled easy retrieval and tracking of the raw data back to the original sources [54]. The researcher’s PhD supervisory team reviewed the data analyses periodically to discuss the analytical choices made.

### Ethical considerations

Respect for persons, beneficence and justice are key principles underpinning ethical research [55]. University of Plymouth ethics approval to undertake the study was granted (reference number 12/13–130). The study did not require full NHS ethical approval at the time it was conducted (2013–14) but required local NHS Research and Development permissions from the 3 NHS Trusts where the focus groups were held, and these were obtained. The participants received a participant information sheet and a covering letter inviting them to attend. Participants were informed that they would be able to contact the researcher for further information or clarification at any time throughout the study phases, which maintained the dynamic process of informed consent. Signed consent was obtained from all participants.

## Results

A total of 34 midwives participated in the focus groups. Ten of the midwives had no previous nursing experience, whilst 24 had trained as nurses before becoming midwives. Sixteen of the midwives identified they had received education and training relevant to OHDC which included ‘in house’ training, university critical care / recovery courses (or similar) and one day training courses, whilst some reported experiential learning.

Framework analysis resulted in five themes, representing the factors influencing midwives to provide OHDC or request a woman’s care be escalated; the environment, maternal wellbeing, fetal / neonatal considerations, the care plan and variable factors encompassing the Delivery Suite workload / staffing and the multidisciplinary team working and support. Notations for data excerpts follow the sequence of maternity unit code / Individual Data (ID) or Focus Group data (FG) / Band of Midwife / Scenario number i.e. S1, S2 or S3 and P represents the participant number.

### The environment

Factors relating to the care environment were ‘fixed’ in as much as the midwives had limited or no opportunity to change them and these included the facilities on the Delivery Suite; dedicated high dependency rooms, equipment and the proximity of the Delivery Suite in relation to the ICU. The bed availability on the ICU or other specialist units such as the coronary care unit also had an impact on the midwives’ decision-making.

Unit H had no designated high dependency beds and the midwives identified they did not have direct access to any of the equipment required for OHDC provision.

*P1: “If they want all that high-tech stuff, we’re not geared up for it here”.*

***(Unit H / FG / Band 6 / S3)***

By contrast, Unit J had a fully equipped, designated high dependency care room on the Delivery Suite with the requisite equipment for midwives to undertake continuous ECG and invasive monitoring. Unit I had a number of large rooms on the Delivery Suite where women could receive OHDC because ‘emergency’ and ‘high dependency’ trolleys carrying the relevant equipment were taken direct to the bedside.

In terms of the location of the Delivery Suite, the midwives from Unit J viewed the very close proximity of the Delivery Suite to the ICU as a positive factor when making escalation decisions. They identified they could accommodate acutely ill women ‘longer’ on the Delivery Suite, knowing the ICU team could provide support very quickly if required. By contrast, the midwives from Unit H identified that the location of their OU (a separate building from the onsite ICU), needed factoring into their EoC decisions.

*P1: “Yeah location wise geography wise here, we’re in an entirely separate entity from the main hospital so that to me, when I’m making decisions makes a difference because you’ve gotta think about a time scale, if you’re asking for help how long its gonna to take to get them there you know and if you want emergency help.”* ***(FG / Unit H / Band 7 / S1)***

Unit I was ‘linked’ to the main general hospital by a series of long corridors, but was a significant distance away from the ICU / general HDU and some midwives identified that the physiological instability of a woman might outweigh the need for her care to be escalated;

*P2: ‘I’d keep her on Delivery Suite, yeah … ..*

*P1: Yeah, it would be … . she's too poorly to be transferred … .*

*P3: “She is, and you wouldn't want to transfer her that ill”.*

***(FG/ Unit I / Band 7/ S1)***

The midwives from Unit H involved the bed manager early on in their care escalation decisions, recognising that ICU beds were not always available and comprised an obstacle to EoC. The midwives from Unit J stated they sometimes gave advance warning to the ICU in order for them to ‘free up’ a bed space in case it became necessary to escalate a woman’s care. The Band 7 midwives of Unit I described how level 3 care (advanced respiratory support) could be provided in the obstetric operating theatre as a temporary measure whilst a bed became available on the ICU.

### Maternal factors

Maternal factors encompassed a woman’s diagnosis, her clinical stability / risk of physiological deterioration and overarching risk status. The midwives’ familiarity with what they identified as routine obstetric complications during the first and second scenarios meant they were more likely to consider providing OHDC.

***Scenario one: What would you do in terms of care escalation?*** *Keep on HDU - 1 midwife. Continue monitoring. As IV [intravenous] MgSO4 [magnesium sulphate] and labetalol, obs [ervations] graduating to at least hourly. 1 hourly reflexes. 6 hourly bloods. Reg [istrar] reviews / consultant as needed.*

***Why?*** *Obstetric care that we deal with regularly.* ***(Unit J / ID / P4 / S1)***

By contrast, Scenario 3 prompted the midwives from all of the OUs to consider the differential diagnoses for the woman, with many of midwives identifying they required a definitive diagnosis in order to determine whether escalation was required or whether care could continue on the Delivery Suite.

*P1: “I mean they usually do a VQ [ventilation perfusion] scan don’t they, that would be fine as far as we are concerned, they go from (name of ward) for a VQ scan and things like that but it’s just that, get a diagnosis first and then decide*”. **(Unit J / FG / Band 6 /S3)**

The midwives identified they were less familiar caring for women with cardiac conditions and there was general agreement during the focus groups that when a woman with a comorbidity had an otherwise uncomplicated pregnancy she should be transferred away from the OU to an appropriate specialist area.

Clinical stability and / or the risk of physiological deterioration featured heavily in the midwives’ EoC discussions. The midwives discussed the objective measures that enabled them to assess a woman’s level of clinical stability and her risk of physiological deterioration, referring to biochemical and haematology results, fluid balance, and MEOWS scores. During Scenario 1, the midwives expressed concerns about the woman’s severely deranged liver function tests, her uncontrolled hypertension and the presence of clonus. They acknowledged the potential for the development of eclampsia, liver haematoma, renal failure and intracranial haemorrhage. The midwives’ predictions as to the type, and likelihood of such complications occurring appeared to influence their EoC decisions. Where physiological instability was evident or the potential for further deterioration was assessed as high, EoC was considered.

Scenario 2 aroused concerns that the woman might suffer further deterioration in the form of haemorrhage, disseminated intravascular coagulation (DIC) and / or fluid overload.

*“Concerns EBL [estimated blood loss] 3000mls, difficult peripheral cannulation, urine output 20mls/ hour. Unstable blood picture. Very high risk, plus high risk of further PPH. DIC. HDU / ICU”* **(Unit I / ID / Band 6 / S2 / P2)**

In conjunction with the objective parameters used to predict a woman’s potential for physiological deterioration some midwives made ‘intuitive’ assessments about a woman’s risk of deteriorating, often using colloquialisms.

***P1*** *: "Yeah – she could have cardiomyopathy.*

***P2*** *: She’s a hot potato. She could explode at any time. P3: Because we haven't got a diagnosis, have we?*

***P1****: No …* ..

***P2*** *: And therein lies the problem … .*

***P3*** *: “Yeah, she could do anything”.*

***(Unit I / FG/ Band 7/ S3)***

The midwives frequently gauged the overall risk status of the women in the scenarios, classing them as being high, very or extremely high risk. They often referred to the type of care the women required as being high risk or complex. These subjective risk estimates appeared to influence their decisions to escalate care to the ICU.

***What would you do in terms of care escalation?*** *..... High risk care not for us. Over 1.5 litres MOHP [Major Obstetric Haemorrhage protocol]* ***Why?*** *Very high risk.* **(Unit H / ID / Band 6 / S2 / P3)**

### Fetal / neonatal considerations

The assessment of fetal wellbeing, either by external CTG and / or ultrasound scan formed an integral part of the care escalation decisions made by the midwives during S3. For some, when ‘obstetric’ complications could be excluded and fetal wellbeing confirmed, escalation of care could be initiated, enabling specialists to manage a woman’s comorbidities.

*P7: “Despite being pregnant she’s not an obstetric case, we’re not concerned about her from an obstetric point of view”.* **(Unit J /FG /Band 7/ S3)**

However, the midwives of Units I and J also discussed how women with comorbidities sometimes remained on the Delivery Suite ‘by default’ because other specialist areas were reluctant to provide care for pregnant women. They described how pregnancy effectively became a barrier to care escalation.

*P3: “ … .She’s got this differential diagnosis of PE or … (pause), but she would have come to us because this happens a lot … as soon as they’ve got that pregnancy they want us to have them”.* ***(FG /Unit J / Band 6 / S3)***

Some midwives aimed to avoid separating mother and baby but were careful to take additional actions to maintain maternal safety, such as ensuring there were adequate staffing levels and appropriate external support for OHDC to be provided.

***What would you do in terms of care escalation?*** *Call critical care team for advice re care of CVP line. Ensure venflon access with team. To stay on HDU Labour Ward.* ***Why?*** *To enable her to be supported to stay with baby, but ensure adequate staff to provide care.* **(Unit J / ID / Band 7 / S2 / P6)**

The midwives appeared to have a lower threshold for escalating a woman’s care away from the Delivery Suite if the neonate had already been separated from her.

***P1*** *: “I think if she’s not got a baby with her, that would be one of the reasons why I would be less inclined to keep her on labour ward because I think to myself well, go let her get well, and then come back and worry about the baby side of things.”*

***P4*** *: “Because if you’ve got a term baby or a baby with her you tend to be trying to keep them together don’t you”.* **(Unit H / FG Band 7 / S1)**

Neonatal gestation was a consideration for the midwives of Unit H who acknowledged that those born less than 30 weeks gestation required transfer away from their OU, as they did not meet the criteria for admission to the onsite SCBU.

### Plan of care

The plan of care included the vigilance and interventions required by a woman and the influence of clinical guidelines. Vigilance encompassed the staff to woman ratios needed for safe OHDC provision and the frequency and type of monitoring required. The midwives agreed that the women in the three scenarios required one to one care, with some specifying a midwife should be in constant attendance. During Scenario 1, the recording of maternal observations every 5 min was accepted by the majority of midwives as suitable for OHDC, with the caveat that there were sufficient staff available. Only one participant questioned whether a woman requiring this frequency of observations required escalation to the ICU.

The midwives’ competence to care for women requiring non-invasive monitoring in the form of a continuous ECG and invasive monitoring (CVP lines) were factors that significantly influenced their decisions to request EoC. There was disagreement between the Band 6 and 7 midwives from Unit J as the Band 6 midwives felt a woman with a CVP line should be transferred to the general HDU in accordance with local guidelines. Conversely, the Band 7 midwives acknowledged the difficulties in midwives maintaining their competencies when managing CVP lines, but discussed strategies to support the Band 6 midwives, who often provided the ‘hands on’ OHDC. These strategies varied, but included involving the theatre / recovery team, CCOT and / or the anaesthetist, to provide the necessary support and education for the midwife allocated to provide the OHDC.

*P6: “She is quite stable, I would probably keep her on HDU on labour ward, but there are a few things I would need to consider … It would have to be a midwife that could do CVP lines, not all our midwives do, umm, however we do work closely with the theatre team and there may be an ODP [Operating Department Practitioner] that can assist us with that.”* **(Unit J / FG / Band 7 / S2)**

The midwives of Unit H, the smallest OU, were united in their decisions to swiftly escalate the care of the woman in Scenario 2. These midwives proactively involved the bed manager, CCOT and ICU staff early on in the scenario and stressed they did not provide care for women with CVP lines. They did not possess the requisite skills (or equipment), and acted in accordance with a local clinical guideline.

*P1: "The minute you said CVP (CVP said in unison by all midwives with some laughter), the lady needs to go!*

*P6: Yeah, we* ***don’t*** *keep her on labour ward.*

*Participants: Yeah." (All agreeing together with some laughter)*

**(Unit H / FG / Band 7 / S2)**

The Band 7 midwives from Unit I spoke about ensuring a midwife ‘experienced’ in managing a CVP line was allocated to care for the woman. They did not elaborate on how they classed a midwife as being ‘experienced’ but acknowledged that they did not encounter women with CVP lines regularly. Enlisting support from the anaesthetist was seen as a strategy for ensuring the midwife allocated to care for the woman was ‘comfortable’ with the CVP line. Moreover, some midwives stated they would provide OHDC if another professional took responsibility for managing the CVP line aspect of care. Midwives stressed that despite once being competent to care for women needing invasive monitoring, they could not keep up to date, as they did not encounter women requiring invasive monitoring on a regular basis.

The request for continuous ECG monitoring during Scenario 3 raised concerns for the majority of midwives who identified they were unable to interpret ECGs. The way midwives dealt with the request for continuous ECG monitoring varied across the OUs but they acknowledged their limitations and took measures to ensure they worked within their professional code. The midwives from OU H identified that assessment by the CCOT would be required with a view to escalating care away from the Delivery Suite.

*P4: " … they also want a continuous ECG, which none of us interpret, we don’t do ECGs …*

*P1: We don’t, we can’t tell when it’s abnormal … ..*

*P2: But we would often have somebody particularly on the [name of ward] ward, we've had people who've had ECGs … ..*

*P1: Yeah, but not continuously … ..*

*P5: If it went beep or something, we'd know it was doing something (laughter) … .. P5: Again outreach would need to be contacted and come and assess."*

**(Unit H / FG/ Band 7 / S3)**

The decision as to whether the woman in Scenario 3 stayed on the labour ward or her care be escalated varied, with some midwives electing to provide OHDC, whilst others identified that transfer to a medical ward or Coronary Care Unit (CCU) was more appropriate. Some of the Band 6 midwives looked to the Critical Care Outreach Team for support.

*P8: " … .but it’s not our speciality to read an ECG … ..*

*P5: But the registrar and the anaesthetist would be coming in to view it. As long as you know what normal is then as soon as you get something strange you get someone to come and review it don’t you."* **(Unit J / FG/ Band 7 / S3)**

The majority of Band 6 and 7 midwives from Unit I stated they would keep the woman on the Delivery Suite. The Band 7 midwives stated they would ask an anaesthetist to interpret the ECG whilst the Band 6 midwives did not have clear strategies for dealing with this but expected another professional to take responsibility for this aspect of the woman’s care.

*P2: "But I agree the scenario is pointing to a PE because she's at a higher risk and I would be happy to keep her as long as they're not relying on me to read that ECG.*

*P3: I don’t think they would … .*

*P2: No … … .*

*P3: I'd expect someone to be in there reviewing it frequently."* **(Unit I / FG / Band 6 / S3)**

The midwives from Units J and I made recommendations for the investigations the women in the three scenarios required. Midwives requested specific investigations to enable them to assess (in conjunction with the MEOWS scoring) whether a woman’s condition was improving or deteriorating. These investigations informed their decisions as to whether they could provide OHDC or needed to escalate care.

*P2: "And do those bloods again to see if that trend is still … ..*

*P1: Yeah we know that those platelets could be plummeting or coming back up … …*

*P3: Get the next lot of bloods really and review, cause if the platelets go lower we’re in trouble".* ***(Unit J / FG / Band 6 / S1)***

The midwives assessed the interventions the women in the three scenarios had received, were receiving and those they might require prospectively, in order to promote physiological stability and avoid escalation of care. During Scenario 1, the midwives discussed the need to increase the woman’s intravenous antihypertensive dose, whilst some considered the need for the addition of a second antihypertensive. They discussed the importance of continuing the magnesium sulphate infusion and ensuring strict fluid restrictions were in place. These measures focused on preventing further physiological deterioration and morbidity / mortality associated with uncontrolled hypertension and fluid overload, thereby negating the need to escalate care away from the Delivery Suite.

*P2: “I'm gonna make sure that lady has one to one care, so her midwife is not needed elsewhere and she hasn’t responded as yet to the Labetalol, her blood pressure is still the same so we could look at what other antihypertensive she could have but you've got to be cautious in case it [BP] crashes.”*

*P3: But equally we know that that there is an increased risk of intracranial haemorrhage if you don’t get their blood pressure down particularly if she’s complaining about a frontal headache.* ***(FG / Unit H /Band 7 /S1)***

Scenario 2 prompted less discussion about prospective treatments from the Band 6 midwives of Unit J and the Band 6 and 7 midwives of Unit H, where the emphasis centred on the issue of the woman having a CVP line in situ and the need for escalation away from the Delivery Suite. In contrast, the midwives of Unit I and the Band 7 midwives of Unit J discussed the need for administration of additional blood products and uterotonics, including tranexamic acid and misoprostol to promote stability and negate escalation. The insertion of a Bakri Balloon to treat / prevent further uterine atony was also suggested.

During Scenario 3, the midwives identified the need for the woman to receive treatment for venous thromboembolism and receive medical input for the management of her diabetes. Whilst the majority of midwives acknowledged the woman required facial oxygen to maintain normal oxygen saturations, greater emphasis was placed on the investigations she required in order to secure a diagnosis and the request for her to receive continuous ECG monitoring.

The midwives used their knowledge of local clinical guidelines to validate their EoC decisions during some, but not all of the discussions. The Band 6 midwives of OU J referred to their escalation policy in relation to scenario one, although some of the Band 7 midwives favoured providing OHDC.

*P3: “The intensivists, that’s in the escalation policy now. If they’ve had an abnormal blood picture they would be speaking with them just to give them the heads up so that they know the blood picture is abnormal. So if she does then require to go over [to ICU], one of our criteria is severe HELLP or she needs a CVP line or art line monitoring, we can’t keep her on the labour ward with those kind of things”.* ***(Unit J / FG /Band 6 /S1)***

The OU H midwives worked to a guideline outlining the specific circumstances when a woman’s care should be escalated to ICU and both Band 6 and 7 midwives appeared to adhere to it. Major obstetric haemorrhage and pre-eclampsia clinical guidelines were recognised and referred to during some of the focus group discussions, especially by the midwives from OU I. Clinical guidelines were not recognised or discussed during the scenario 3 focus groups, suggesting there were no specific guidelines available to influence the midwives’ decision making when faced with this type of clinical scenario.

### Staffing levels, skill mix and workload

The Delivery Suite staffing levels, midwifery skill mix and workload were ‘variable factors’ considered by the midwives when deciding whether OHDC could be provided. The importance of being able to provide skilled, one to one care was often emphasised.

*P6: “Staffing, because I notice the staffing and the ward is very busy, so I would need to risk assess because obviously, she needs one to one care. It would have to be a midwife that could do CVP lines, not all our midwives do … .I would also be looking at do I need to get more staff to help cover labour ward because I have to look at the risks on the labour ward with such a sick patient around … .”* ***(Unit J / FG / Band 7 / S2)***

Skill mix in the context of midwives providing OHDC appeared to be synonymous with those classed as experienced, senior or competent. Midwives who had undertaken registered nurse training were viewed positively with regards OHDC provision, although it was acknowledged that a nurse qualification did not take the place of ongoing OHDC education and training.

*P4: “A good thing to have is a nurse who’s then become a midwife, and that’s a good background for caring for somebody who is this ill, but only in as much as you want HDU training for your midwives because that’s what we’ve not got, because I don’t have that. It takes all my efforts to become a bit nursey again and work out the nursing side of it, I could do the midwifery side of it until it gets very very abnormal”.* ***(Unit I / FG/ Band 6 / S1)***

The Band 7 midwives of Unit J suggested that skill mix was at times, more important than adequate staffing levels, when women required OHDC.

*P3: “I know we always talk about staffing and things but I do feel it’s more skills than numbers, we have lots of conversations about this don’t we? You could have ten midwives on duty but no one able to look after this sick lady. Whereas you could have five midwives on duty and any one of them could look after this kind of patient, so I think it is definitely about skills and abilities as well as numbers.”* ***(Unit J / FG /Band 7 /S2)***

The lack of (and need for) adequate training to enable midwives to provide OHDC was raised by some of the focus group participants who expressed concerns that the differing abilities of the midwives to provide care for sick women, had the potential to lead to inequalities in care provision.

*P3: “It’s not fair on the women to have inequality of care because somebody might have HDU and critical care skills and be quite happy to do it but the next person isn’t, so you’ve got to have some kind of policy to escalate the women’s care so that they’ve got equality of care in the appropriate place”.*

***(FG/ Unit I / Band 6 / S1)***

Workload also had an impact on the midwives’ EoC decisions. High activity levels and reduced staffing on the Delivery Suite were considered as triggers for escalation. Some midwives stated they would instigate the staffing escalation guideline so that OHDC could be provided.

### Multidisciplinary team working and support

The presence of, and support from the multidisciplinary team had a vital impact on midwives’ decisions to provide OHDC. Support came from internal and external sources. Internal supporters were those professionals who worked permanently or frequently within the OU setting and included the midwife in charge of the Delivery Suite (the co-ordinator), Consultant Obstetricians and Anaesthetists, Operating Theatre Team / ODP and Registered General Nurses (in one OU only).

The Band 6 midwives identified they would seek advice and guidance from the co-ordinators and involved them early in the decision making process as to whether OHDC could be provided. The co-ordinators were recognised for their experience and clinical expertise. Some Band 7 midwives stressed the importance of physically reviewing a woman, and not relying on verbal information alone. They appeared to use their clinical expertise and intuition to assist them in gauging the severity of a woman’s condition.

*P3: So any woman who is critically ill (long pause) then somebody like this needs to be physically reviewed by the coordinator. It's no good having the story at the board. You need to go in and see them.*

*P1: Yeah, definitely.*

*P2: Because your experience and instinct will tell you just how well or how unwell she is.* ***(Unit I /FG/ Band 7 /S3)***

The midwives also looked to the senior obstetricians and anaesthetists for support in ensuring safe OHDC could be provided. Anaesthetists were identified as the professionals who supported the midwives with ECG interpretation, the management of invasive monitoring and providing general advice regarding the care required by critically ill women. Additional internal support for women requiring invasive monitoring was sought from the theatre team and / or the Operating Department Practitioners (ODPs).

*P7: “Recently I got theatre, because we were busy on labour ward and the midwife didn’t know how to use the CVP line and the theatre staff came across and ran thorough it with her, and next time they watched her do it and she was absolutely fine”.* ***(Unit J /FG/ Band 7 /S3)***

The Band 7 midwives from Unit I recognised the contribution made by the nurses who were sometimes allocated to provide OHDC. The Band 6 midwives seemed less positive about the nurses’ involvement.

*P2: “It’s not appropriate that they should be caring for them [Women needing OHDC] because they’ve got no midwifery training, they don’t know the significance, ‘because I’ll just walk in the room and know because with experience, well they haven’t got that, have they?”* ***(FG/Unit I/ Band 6 S1)***

The Band 6 midwives were also concerned they might become deskilled in OHDC provision because increasingly the nurses were allocated to provide this care.

External supporters were those professionals who worked outside of the OU, but were called upon to support staff to provide OHDC or facilitate escalation. External supporters included the Intensivist / ICU nurses, CCOT, Haematologist, Cardiologist, Physicians and Bed Managers. Referral to and liaison with intensivists, the ICU and the CCOT were mentioned the most frequently of all the external supporters available to the midwives from Units H and J.

*P2: If she started to deteriorate any more, if she needed more oxygen and her respiratory rate was going up and you had all the other signs … ..*

*P9: That’s were your outreach comes in … ..*

*P5: and we have called outreach before and they can get them a bed really quickly on ICU.* ***(FG / Unit J / Band 7 / S3)***

The midwives from Unit I appeared to liaise with the consultant anaesthetist as the first line of support more frequently than the CCOT, whilst in Unit H, the midwives looked to the CCOT to provide clinical support, liaise with other professionals and organise transfers from the OU to the ICU or general HDU. The midwives from Unit H were very clear as to who they contacted for OHDC support and when. They worked to a clinical guideline and did not deviate. The focus groups held with the midwives of Units I and J suggested that local variations between midwives working on the same Delivery Suite were sometimes apparent, with different midwives seeking different support mechanisms.

## Discussion

This study highlights the complex interplay of factors that may influence midwives’ decisions when deciding whether to provide OHDC or escalate care. Five key findings that influenced the midwives’ decisions will be discussed and encompass (1) the care environment, (2) the maternal condition, (3) the plan of care for the mother (specifically the need for invasive and / or ECG monitoring), (4) multidisciplinary support for midwives to provide OHDC and (5) the variable factors of midwifery skill mix, staffing levels and workload on the Delivery Suite.

Corroborating the findings of previous studies, with regards the *care environment*, the midwives working in the OU with the lowest annual birth rate did not have the specialist equipment required to provide all aspects of OHDC and identified they would automatically escalate care away from the Delivery Suite when invasive or continuous ECG monitoring was required [9, 45]. The proximity of the Delivery Suite to the ICU also had some influence on the midwives’ EoC decisions. The midwives working on the Delivery Suite situated close to the ICU viewed this as a ‘safety net’ when providing OHDC as they were able to obtain specialist help rapidly if required. Conversely, due to the substantial transfer distance between the onsite ICU and Delivery Suite, the midwives of OU H appeared to make their escalation decisions ‘early’, taking this into consideration. This OU was separate, but on the same site as the ICU, classed as “split site” [56]. Adverse incidents occurring during intra hospital transfer of critically ill adults includes further physiological deterioration, although involvement of the CCOT has been promoted as a measure to enhance maternal safety, and was advocated by some of the midwives in this study [57, 58].

In relation to *maternal factors*, the midwives were confident discussing the care of women who had diagnoses of common obstetric conditions and complications, but were more reluctant to provide care for the woman in the third scenario whose diagnoses was unconfirmed but potentially cardiac in origin. This finding replicates research suggesting doctors are more inclined to escalate care if they are unfamiliar “with a patient’s clinical problem” [59]. Moreover, it is unsurprising that the midwives viewed physiological instability as an indication for escalation given that unresolved physiological instability is associated with increased severity of illness and higher patient acuity, necessitating complex haemodynamic monitoring and more active treatments outside the facilities within OUs [2, 60]. However, the midwives also discussed escalation for clinical deterioration they predicted, based on their clinical intuition; an aspect of practice being researched in other clinical specialities and widely discussed in the literature [61–63].

The majority of the midwives in this study identified they could not interpret an ECG and those who had been trained to care for women with invasive monitoring had become deskilled. Competence is influenced by ‘skills fade’ - a decline in the ability of a practitioner to perform a specific skill over time, if it is not undertaken for long periods [64, 65]. To date, an accepted ‘frequency of exposure’ to women requiring OHDC, that enables midwives to maintain basic levels of competency has not been recommended, and is a subject of debate in relation to patient safety [66]. Given that skills fade is individual specific, it may be suggested that midwives themselves should be encouraged to determine when they require updating, and take the lead in identifying their own learning requirements [64] which, can subsequently be supported at an organizational level by, for example, rotating them into the ICU or general HDU.

Several midwives appeared to use ‘workarounds’ to solve the issue of skills fade / skills deficit, in order to optimise care of the women in the scenarios and negate escalation. Workarounds are described as the “problem solving measures” or “improvisations” used by practitioners for “overcoming a problem or limitation in a system” [66–68]. In the context of this study, the midwives’ workarounds comprised enlisting the support of multidisciplinary team members to assist them in providing the hands on OHDC they could not undertake (i.e. invasive monitoring and ECG interpretation). Anaesthetists have previously been recognised for their supportive role in assisting midwives to provide care for critically ill women [1, 38, 69] as has the CCOT [70, 71]. Interestingly, the midwives in this study showed no reticence in contacting the CCOT, contrary to previous findings [31]. The sources of help enlisted by the midwives working in the two largest OUs tended to vary from midwife to midwife; this has the potential for inconsistency of advice and lack of clarity regarding lines of professional responsibility and accountability. It is advocated that workarounds should not be used as long-term solutions to issues arising in clinical practice as they may lead to patient safety incidents (especially where lines of responsibility are unclear), and resolution at the higher organizational level is required [66, 67, 72].

Cost implications of upskilling midwives to provide OHDC may be justified by the need to provide organisationally robust systems which enhance maternal safety, relieve some pressure on ICUs / general HDUs [16, 73] and proactively maintain the mother and baby relationship [2]. Alternatively, the mobilisation of *formal* support mechanisms into the OU, agreed at local level, may be used to facilitate midwives to provide safe OHDC and negate escalation of women away from the Delivery Suite [8]. These mechanisms may include involvement of the CCOT, recovery nurses and ODPs [58, 74] but must be formalised in escalation guidelines / protocols and incorporated into practice in a robust manner, thereby streamlining procedures and negating workarounds.

Anecdotal evidence suggests that a combination of critical care nurses and midwives providing OHDC “has been shown to work well in some units” [75] although this may “labour intensive” [38]. Nurses were involved in OHDC provision in one of the OUs in this study and is an example of ‘task shifting’. Task shifting has been advocated by the King’s Fund as a means of freeing up midwives to provide the care that they alone can provide (e.g. intrapartum care) and has been proposed as a cost effective solution to promoting safe care [76, 77]. However, task shifting may lead to professional tensions [77] as apparent in this study, with the midwives concerned they may become deskilled in OHDC provision.

Midwives also considered escalating care away from the Delivery Suite if the staffing levels and / or skill mix were perceived to be inadequate or the workload judged too high for the numbers of midwives on duty. This finding reflects those of an audit of OUs in the United Kingdom (*n* = 146) which reported staff had “low thresholds” for transferring women to the ICU due to suboptimal staffing “skill levels” [33]. This course of action supports safe practice considering other studies have reported that inadequate skill mix and suboptimal staffing levels can negatively influence the way that deteriorating patients are managed [26, 30] but may place undue pressure on ICUs and disrupt the mother baby relationship.

### Strengths and limitations

The focus group study successfully used video vignettes in conjunction with objective data to mimic real life clinical scenarios and trigger midwives’ discussions related to their EoC decisions. This method was chosen as ethnographic observation was deemed ethically inappropriate and logistically problematic. None of the scenarios included an acutely ill woman in labour and this is a study limitation. A proportion of women will require OHDC during the intrapartum period [6] and the introduction of this type of scenario may have provided further insight into this aspect of practice. Given the importance of multidisciplinary team working in OHDC provision [11], the exclusion of obstetricians, anaesthetists and critical care staff from the focus groups may also be viewed as a study limitation; involvement of these professionals would have provided additional, valuable viewpoints.

## Conclusion

Midwives working in the OU with the lowest annual birth rate did not have access to the equipment needed to provide OHDC and requested care be escalated away from the OU, confirming previous propositions in the published literature. Some, but not all midwives from the two larger OUs were willing to provide OHDC, but their decisions were influenced by a combination of clinical and professional factors. The absence of a definitive diagnosis, unfamiliarity with a condition and physiological instability increased the likelihood of midwives considering a woman’s care needed escalation to the ICU. Midwives from the larger OUs were more likely to provide OHDC, but did not always possess the skills to do so and used workarounds to resolve their deficits, by seeking support from other members of the multidisciplinary team. Some midwives considered escalating care away from the OU when skill mix and workload were deemed suboptimal for safe OHDC provision and this aspect of care lends itself to local auditing. Safe OHDC provision requires organisationally robust systems that ensure midwives are not required to use workarounds, and have access to guidelines that take into consideration the impact of the changing workload on the Delivery Suite. Formal education and training strategies for midwives providing OHDC in DGHs where women requiring this type of care are not encountered regularly must include strategies to negate skills fade.

## Data Availability

The raw / analysed qualitative data generated during the study will not be made publicly available as it has the potential to identify individual maternity units and anonymity of the research sites and midwives involved must be protected.

## Abbreviations

CCOT: Critical Care Outreach Team
CTG: Cardiotocograph
CVP: Central Venous Pressure
DIC: Disseminated Intravascular Coagulation
EBL: Estimated Blood Loss
ECG: Electrocardiogram
EoC: Escalation of Care
HDU: High Dependency Unit
HELLP: Haemolysis, Elevated Liver enzymes, Low Platelets
ICU: Intensive Care Unit
I-CVI: Item-Content Validity Index
LNU: Local Neonatal Unit
MEOWS: Modified Early Obstetric Warning Systems
NHS: National Health Service
NICU: Neonatal Intensive Care Unit
NMC: Nursing and Midwifery Council
ODP: Operating Department Practitioner
OHDC: Obstetric High Dependency Care
OU: Obstetric Unit
RRS: Rapid Response Systems
SCBU: Special Care Baby Unit
TTS: Track and Trigger System
